# Prognostic significance of intermediate and high SUVmax values and imaging timing on FDG-PET/CT after radiation therapy in HNSCC: a retrospective cohort study

**DOI:** 10.3389/fonc.2026.1923156

**Published:** 2026-09-16

**Authors:** Thomas Hebisch, Kerem Tuna Tas, Philipp Lishewski, Fatima Frosan Sheikhzadeh, Klemens Zink, Boris A. Stuck, Hilke Vorwerk, Sebastian Adeberg, Ahmed Gawish

**Affiliations:** 1 Department of Radiotherapy and Radiation Oncology, Marburg University Hospital, Marburg, Germany; 2 Department of Radiotherapy and Radiation Oncology, Philipps-Universität Marburg, Marburg, Germany; 3 Marburg Ion-Beam Therapy Center (MIT), Department of Radiotherapy and Radiation Oncology, Marburg University Hospital, Marburg, Germany; 4 University Cancer Center (UCT) Frankfurt - Marburg, Marburg, Germany; 5 LOEWE Research Cluster for Advanced Medical Physics in Imaging and Therapy (ADMIT), Technische Hochschule (TH) Mittelhessen University of Applied Sciences, Giessen, Germany; 6 Department of Otorhinolaryngology, Head and Neck Surgery, University Hospital Marburg, Philipps-Universität Marburg, Marburg, Germany

**Keywords:** chemoradiation, head and neck cancer, PET, progression, radiotherapy, SUV, FDG

## Abstract

**Introduction:**

Head and neck cancer is a disease with a tendency for recurrence and metastases even after treatment. Late detection leads to poor patient outcomes. One promising diagnostic tool is FDG-PET/CT which can detect both, morphological and metabolic evidence of malignant diseases. A common measure is SUVmax, the maximum standardized uptake value in a lesion. The objective of this study is to evaluate the prognostic value of post-treatment FDG-PET/CT in patients with head and neck squamous cell carcinoma (HNSCC), focusing on the association of SUVmax and scan timing with progression-free survival (PFS).

**Methods:**

This retrospective study included 84 patients with HNSCC treated with definitive radiotherapy between 2016 and 2023 at a German university hospital. All patients underwent at least one post-radiotherapy FDG-PET/CT scan. All post radiation scans that were performed before or no later than one month after documented disease progression were included for prognostic analysis. SUVmax values were categorized into three groups: (1) physiological uptake (no suspicious lesion, no SUVmax value recorded, and radiological report consistent with post-therapeutic changes), (2) intermediate uptake (SUVmax >1 but <7.5), and (3) high uptake (SUVmax ≥7.5). PET/CT timing was stratified as early (≤3 months) or delayed (>3 months) after radiotherapy. Diagnostic performance metrics and survival outcomes were analyzed.

**Results:**

A total of 88 post-treatment PET/CT scans were evaluated. Mean follow up was 27.7 months. Disease progression occurred in 33 patients and 10 patients died during follow-up. SUVmax ≥7.5 was significantly associated with shorter PFS (p < 0.05). Intermediate SUVmax values also predicted increased progression risk compared to physiological uptake. The optimal diagnostic performance was achieved with delayed PET/CT (>3 months), particularly using a low SUVmax threshold (>1), which yielded high sensitivity (90.9%) and negative predictive value (88.9%). Multivariate analysis confirmed SUVmax as an independent predictor of PFS (HR: 1.21; 95% CI: 1.11-1.32; p < 0.001), alongside male sex and oral cavity primary site.

**Conclusion:**

Post-treatment FDG-PET/CT is a valuable prognostic tool in HNSCC follow-up. SUVmax, especially when evaluated after three months, is predictive of disease progression. Integrating PET/CT into routine surveillance may improve risk stratification and inform tailored follow-up intensity.

## Introduction

Head and neck squamous cell carcinoma (HNSCC) represents a significant global health challenge, with an estimated 900, 000 new cases and over 450, 000 deaths annually worldwide (1). The disease is characterized by its aggressive nature and propensity for local recurrence and distant metastasis, making early detection and effective treatment critical for improving patient outcomes (2). Definitive chemoradiotherapy (CRT) is a cornerstone of treatment for locally advanced HNSCC, offering curative potential while preserving organ function (3). However, treatment response varies widely among patients and the ability to accurately assess treatment efficacy and predict outcomes remains a clinical challenge. Salvage surgery, for example salvage neck-dissection, in case of initial treatment failure can expose patients to associated risks and potential complications.

18-Fluorodeoxyglucose (18F-FDG) positron emission tomography combined with computed tomography (FDG-PET/CT) has emerged as a powerful imaging modality for evaluating treatment response in HNSCC. FDG-PET/CT provides both anatomical and metabolic information, allowing for the detection of residual or recurrent disease with high sensitivity and specificity (**Figure 1**) (4). While volumetric measurements like total lesion glycolysis (TLG) are being investigated they are not commonly used outside of study environments and offer drawbacks such as lower inter-reader reliability and the technique of the measurement being more complex (5). The maximum standardized uptake value (SUVmax), a semi-quantitative measure of the highest metabolic activity voxel in an area, is very commonly used to differentiate between post-treatment inflammation and residual tumor (6). Additionally, the timing of PET/CT after CRT may influence its predictive accuracy, as early scans can be confounded by treatment-related inflammation (7, 8).

**Figure 1:**
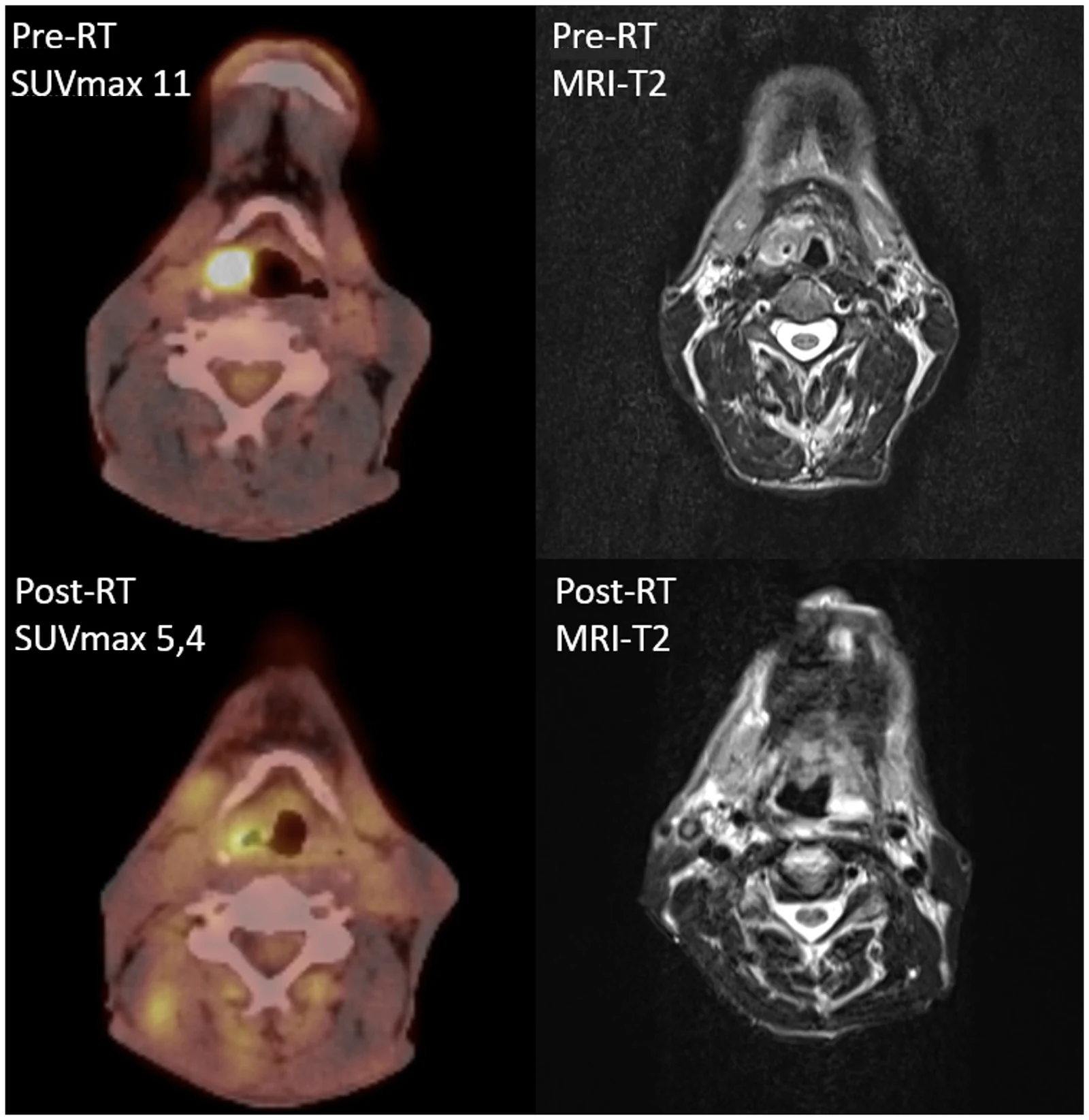
Pre- and post-radiation therapy (RT) PET/CT and MRI of one head and neck squamous cell carcinoma patient.

Despite the growing use of FDG-PET/CT in HNSCC, questions remain regarding its optimal application in clinical practice. Specifically, the prognostic significance of SUVmax, especially equivocal values, and the ideal timing of post-treatment imaging are areas of ongoing investigation. This study aimed to evaluate the prognostic value of post-treatment FDG-PET/CT in HNSCC patients, focusing on the impact of SUVmax on progression-free survival (PFS) and the impact of the timing of imaging on the diagnostic parameters sensitivity, specificity, positive predictive value, and negative predictive value.

## Materials and methods

### Study design and patient population

Following approval by the institutional ethics committee, we conducted a retrospective single-institution cohort study at a university hospital in Germany. The study included patients with histologically confirmed squamous cell carcinoma (SCC) of the nasal cavity, nasopharynx, oropharynx, hypopharynx, larynx, or oral cavity who received definitive radiotherapy, with or without concurrent systemic therapy, between 2016 and 2023.

Exclusion criteria comprised absence of post-radiation FDG-PET/CT imaging, palliative treatment intent, patients under 18 years of age or pregnant at the time of treatment, and lastly absence of at least one FDG-PET/CT image that was taken before or at the latest one month after clinical, radiological, or histological diagnosis of SCC recurrence or progression in order to rule out that the findings reflect the result of the salvage therapy rather than the recurrence or progression itself. Of 652 patients screened, 84 met all inclusion criteria and none of the exclusion criteria.

The study was approved by the institutional review board, and the requirement for informed consent was waived due to its retrospective nature.

### Treatment protocols

Treatment recommendations were made by a multidisciplinary tumor board following initial diagnostic work-up. Diagnostic staging included clinical examination, cross-sectional imaging (CT and/or MRI), and FDG-PET/CT in selected cases. Histologic confirmation was obtained via biopsy or surgical specimen review. Histopathological evaluation was performed by board-certified pathologists.

Radiotherapy was delivered with curative intent using linear accelerator-based photon therapy. The most commonly used regimen was 67.5 Gy in 30 fractions (2.25 Gy per fraction), administered five times per week. Selected patients received a carbon ion or proton boost in combination with photon therapy. Systemic therapy included platinum-based chemotherapy or, in selected cases, checkpoint inhibitor immunotherapy. Treatment was administered either in an inpatient or outpatient setting depending on clinical condition and institutional logistics.

### FDG-PET/CT imaging

All included patients underwent at least one FDG-PET/CT scan after completion of radiotherapy. In-house and externally acquired scans were accepted. For in-house PET/CT, blood glucose was confirmed to be within acceptable limits prior to tracer administration. FDG was administered intravenously with a standard uptake time of approximately 60 minutes. PET/CT image acquisition and reconstruction followed standard institutional protocols.

SUVmax values were measured for all scans by a board-certified nuclear medicine specialist and documented alongside anatomical localization. The documentation also included a short report for the regions head and neck, thorax, abdomen, and extremities with either a described pathology and an SUVmax value or a statement of no suspicious findings. Only documented SUVmax values within the head and neck region were analyzed in this study. In cases of multiple scans, all scans that were performed before or at the latest within one month of recurrence or progression were used as individual data points for progression-free-survival analysis.

### Follow-up

All patients were monitored under structured follow-up programs in both the radiation oncology and head and neck surgery department. The first post-treatment visit occurred approximately six weeks after radiotherapy completion. During the first year, patients were evaluated every three months by both a radiation oncologist and a head and neck surgeon, including cross-sectional imaging (CT, MRI, or PET/CT). Thereafter, follow-up intervals were extended to every six months for four additional years.

In cases of suspected disease progression, patients were re-staged, and management decisions were re-discussed in a multidisciplinary tumor board. Salvage treatment options included re-irradiation, systemic therapy, or surgical intervention, tailored to individual clinical scenarios.

### Statistical analysis

The time interval between completion of radiotherapy and PET/CT imaging was stratified into two groups: early (0–3 months) and delayed (>3 months). SUVmax values were categorized into three groups: (1) physiological (SUVmax assumed to be 1), (2) intermediate (<7.5, but >1), and (3) high (≥7.5). (**Figure 2**) The threshold of 7.5 was chosen according to the median SUVmax value among all non-physiological scans in order to split all scans that were already classified as pathological scans into ‘intermediately pathological’ and ‘highly pathological’ scans relative to our patient population. Physiological scans were defined as those with both no SUVmax value explicitly recorded and no suspicous findings outside of regular and expected post-radiogenic changes reported. If there was an SUVmax value reported the SUVmax value was categorized into one of the groups as seen above.

**Figure 2:**
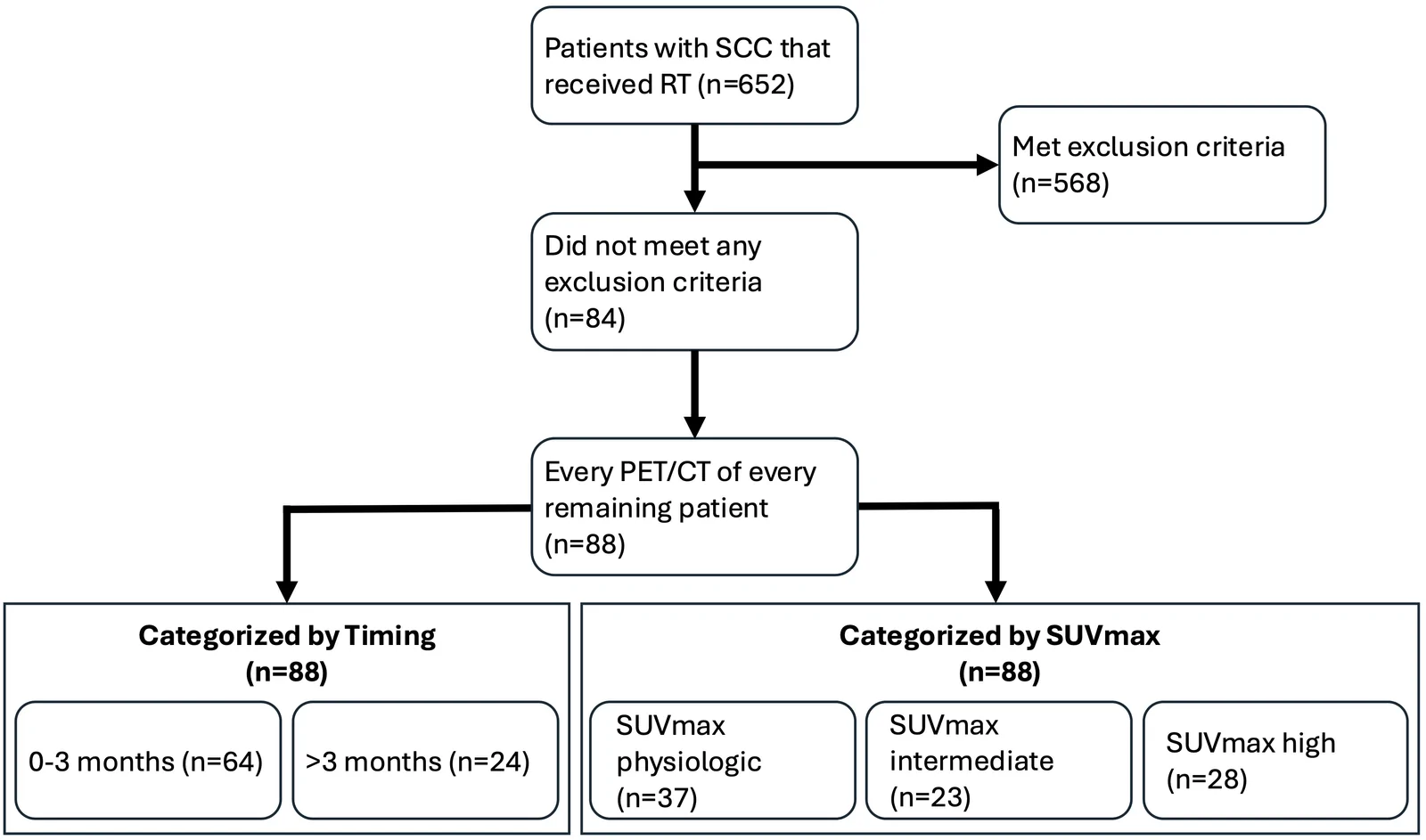
Patient population flow chart.

Descriptive statistics were used to summarize baseline patient and treatment characteristics. Continuous variables are reported as means with standard deviation (SD) and range. Categorical variables are reported as absolute frequencies and percentages.

Diagnostic accuracy metrics (sensitivity, specificity, positive predictive value [PPV], and negative predictive value [NPV]) were calculated using 2×2 contingency tables for selected SUVmax thresholds, both overall and stratified by PET/CT timing (≤3 months vs. >3 months). True progression was defined as any radiologically or histologically confirmed recurrence or increase in TNM stage after radiotherapy completion.

Progression-free survival (PFS) was estimated using Kaplan-Meier analysis, stratified by SUVmax categories and recurrence location (local, regional, distant). Log-rank tests were employed to compare survival distributions. Univariate Cox regression analysis was performed to evaluate the validity of using the assumed SUVmax values in the non-pathological group for prognostic analysis.

Multivariate Cox proportional hazards regression was used to identify independent predictors of PFS. Variables included in the model were SUVmax, human papillomavirus (HPV) status, tumor differentiation, tumor site, age, sex, and PET/CT timing. Statistical significance was defined as a p-value <0.05. All analyses excluding univariate Cox regression analysis were conducted using GraphPad Prism version 10.4.1 (GraphPad Software, Boston, MA, USA). Univariate Cox regression analysis was conducted using GraphPad Prism version 11.0.2 (GraphPad Software, Boston, MA, USA).

## Results

### Patient characteristics

A total of 84 patients with histologically confirmed head and neck squamous cell carcinoma (HNSCC) were included (**Figure 2**). The cohort comprised 65 males (77.4%) and 19 females (22.6%), with a mean age of 60.7 years (SD: 9.5 years). At initial presentation, lymph node metastases were present in 54 patients (64.3%), while distant metastases were identified in 3 patients (3.6%). According to the Union for International Cancer Control (UICC) staging system (9), 1 patient (1.2%) was in stage I, 5 (6.1%) in stage II, 19 (23.2%) in stage III, and 57 (69.5%) in stage IV; staging was inconclusive in 2 cases (2.4%). HPV status was positive in 20 patients (23.8%), negative in 33 (39.3%), and unknown in 31 (36.9%). Most tumors were moderately (n = 43, 51.2%) or poorly differentiated (n = 29, 34.5%). The most common tumor sites were the larynx (n = 34, 40.5%) and oropharynx (n = 24, 28.6%), followed by the hypopharynx (n = 12, 14.3%), nasal cavity and oral cavity (each n = 6, 7.1%), and nasopharynx (n = 2, 2.4%) (**Table 1**).

**Table 1:** Descriptive statistics of patient cohort.

Parameter	Subgroup	Frequency/mean	Percentage/standard deviation with range
Age		60.7 years	9.47 years, 43–92 years
Sex	Male	65	77.4%
	Female	19	22.6%
Stage (UICC)	I	1	1.2%
	II	5	6.1%
	III	19	23.2%
	IV	57	69.5%
	unclear	2	2.4%
HPV Status	positive	20	23.8%
	negative	33	39.3%
	not specified	31	36.9%
Differentiation	well	4	4.8%
	moderate	43	51.2%
	poor	29	34.5%
	undifferentiated	2	2.4%
	not specified	6	7.1%
Location	Nasal cavity	6	7.1%
	Nasopharynx	2	2.4%
	Oral cavity	6	7.1%
	Oropharynx	24	28.6%
	Hypopharynx	12	14.3%
	Larynx	34	40.5%
Therapies	1st Radiotherapy (photons)	84	100%
	Re-irradiation (photons)	9	10.1%
	Particle radiation	11	12.4%
	Chemotherapy	67	79.8%
	Immunotherapy	11	12.4%
	Salvage Neck dissection	16	18.2%
	Non-Neck Dissection salvage surgery	2	2.2%
Progressive Disease Location	Overall	33	39.3%
	Local	14	16.7%
	Regional	13	15.5%
	Distant	9	10.7%
Deaths		10	11.9%
SUVmax Values		4.4	4.6, 1 - 21
Last Follow-Up		27.6 months	24.3 months, 2–139 months
Progression-Free Survival	Overall	21.8 months	23.8 months, 1–139 months
	Local	24.2 months	23.3 months, 2–139 months
	Regional	25.4 months	24.9 months, 1–139 months
	Distant	26.4 months	23.6 months, 2–139 months

### Treatment characteristics

All patients received curatively intended radiotherapy. The most common protocol (n = 51, 60.7%) was 67.5 Gy in 30 fractions (2.25 Gy/fraction, 5 fractions per week). A photon-particle combination boost was administered in 11 cases (12.4%).

Concomitant or sequential chemotherapy was delivered to 67 patients (79.8%), predominantly cisplatin-based. Immunotherapy with checkpoint inhibitors were administered in 11 patients (12.4%). 9 patients (10.1%) underwent photon-based re-irradiation. Surgical interventions included salvage neck dissections (n = 16, 18.2%) and salvage procedures without neck dissection (n = 2, 2.2%). Notably, 18 of 19 patients undergoing any form of post-radiation surgery had post-radiation PET/CT scans with SUVmax >1 (94.7%) (**Table 2**).

**Table 2:** Surgeries after radiotherapy stratified by SUVmax >1/=1.

Stratification criteria	Non-neck dissection salvage surgery	Salvage neck dissection
Absolute frequency SUVmax > 1	2	16
Absolute frequency SUVmax = 1	0	1
Percentage (%) SUVmax > 1	100	94.12
Percentage (%) SUVmax = 1	0	5.88

### Follow-up and outcomes

The median follow-up was 20.3 months (mean: 27.7 months; SD: 23.5 months). Disease progression occurred in 33 patients (39.3%). Among these, local progression was noted in 14 cases (16.7%), regional in 13 (15.5%), and distant progression in 9 (10.7%), with some patients experiencing multiple sites of relapse. A total of 10 patients (11.9%) died during the follow-up period (**Table 1**). A total of 88 FDG-PET/CT scans were performed. Histopathological testing was obtained in 65 cases (73.9%), and malignancy was confirmed in 22 cases (33.8% of all tested cases).

### Influence of SUVmax on PFS and diagnostic performance

In Kaplan-Meier analysis SUVmax values ≥7.5 were significantly associated with increased risk of overall disease progression (p < 0.05). Patients with intermediate SUVmax values (>1 to <7.5) also showed significantly higher overall progression rates compared to the physiological group (SUVmax ≤1) (p < 0.05) (**Figure 3**). This was also the case for each anatomical location individually: for local (in- field) progression higher SUVmax groups were significantly associated with increased risk (p < 0.05) (**Figure 4**), for regional (out-of-field) progression higher SUVmax groups were significantly associated with increased risk (p < 0.05) (**Figure 5**), and for distant progression higher SUVmax groups were significantly associated with increased risk (p < 0.05) (**Figure 6**). A moderate negative correlation was observed between individual SUVmax values and overall progression-free survival (PFS) (Spearman’s ρ =-.35; one-tailed p = 0.0004). Kaplan-Meier analysis did not demonstrate a statistically significant association between SUVmax group and overall survival. Diagnostic performance of different SUVmax thresholds is summarized in **Table 3**. Using a SUVmax cut-off of ≥7.5, sensitivity was 42.4% (95% confidence interval (CI): 27.2-59.2%), specificity 78.2% (95% CI: 65.6-87.1%), PPV 53.9%, and NPV 69.4%. In contrast, a cut-off of >1.0 yielded higher sensitivity (84.9%; 95% CI: 69.1-93.4%) but lower specificity (60.0%; 95% CI: 46.8-71.9%). NPV was higher with the lower cut-off (86.8%), while PPV was comparable (56.0%).

**Figure 3:**
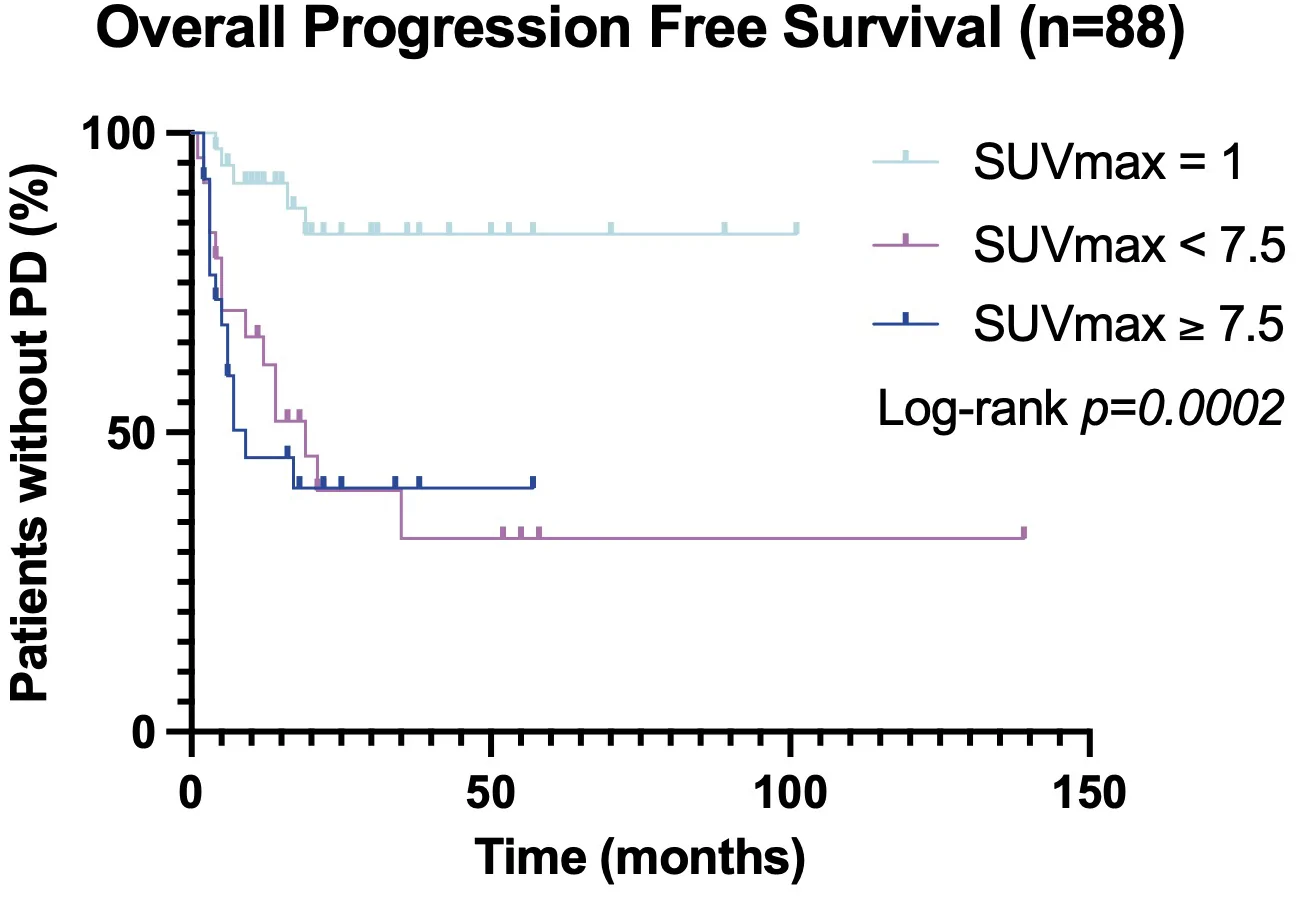
Kaplan-Meier diagram of overall progression free survival for all 88 PET/CT. PFS, progression free survival.

**Figure 4:**
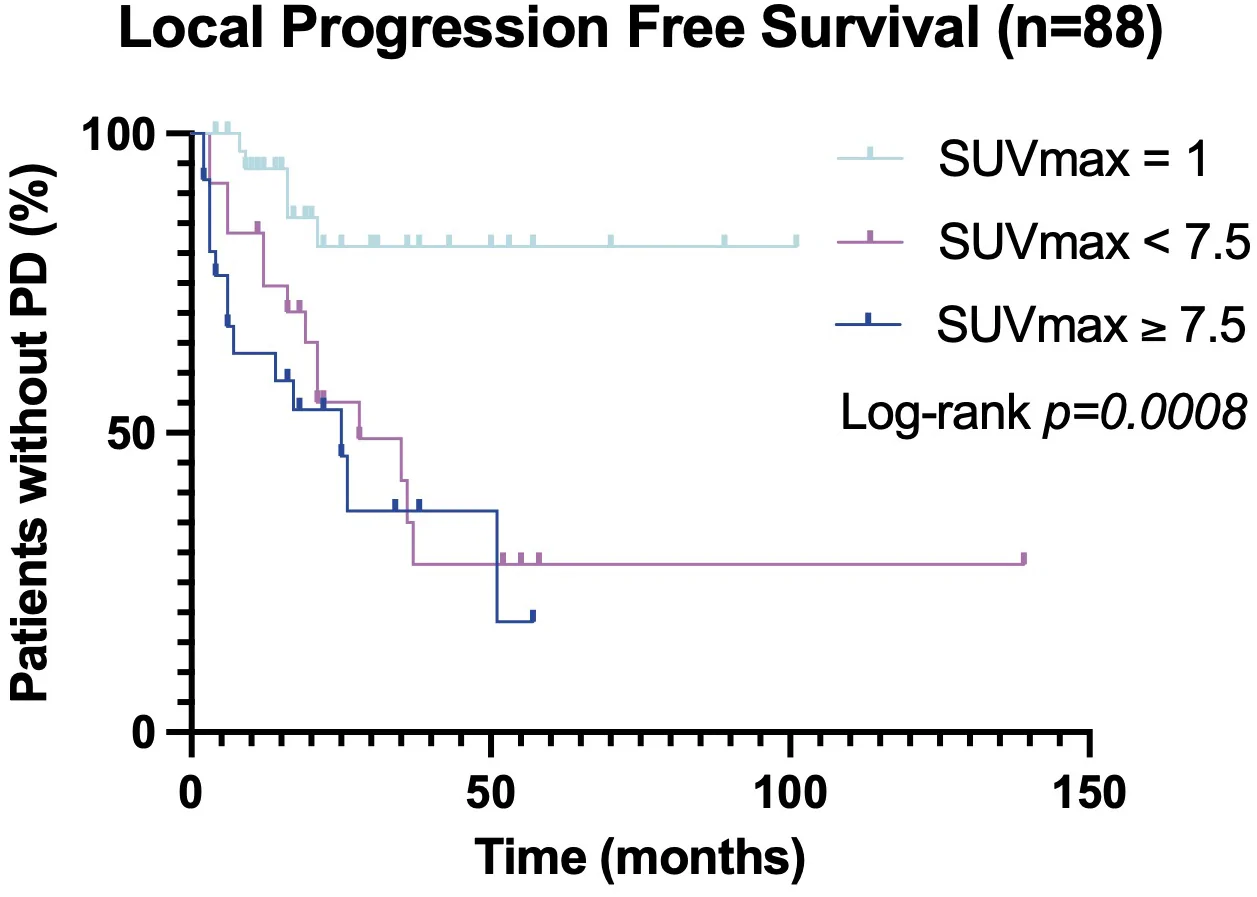
Kaplan-Meier diagram of local (in-field) progression free survival for all 88 PET/CT. PFS, progression free survival.

**Figure 5:**
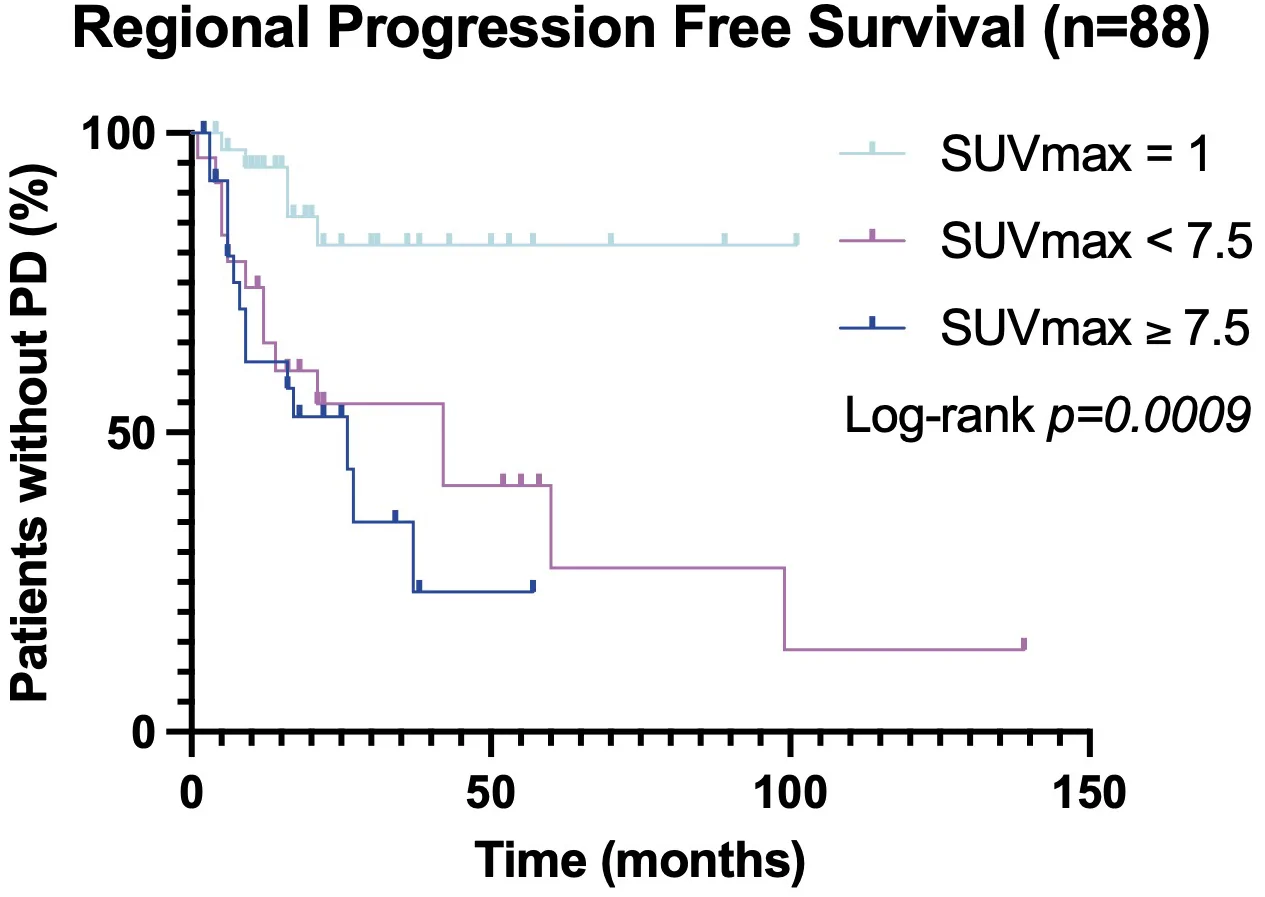
Kaplan-Meier diagram of regional (Out-of-Field) progression free survival for all 88 PET/CT. PFS, progression free survival.

**Figure 6:**
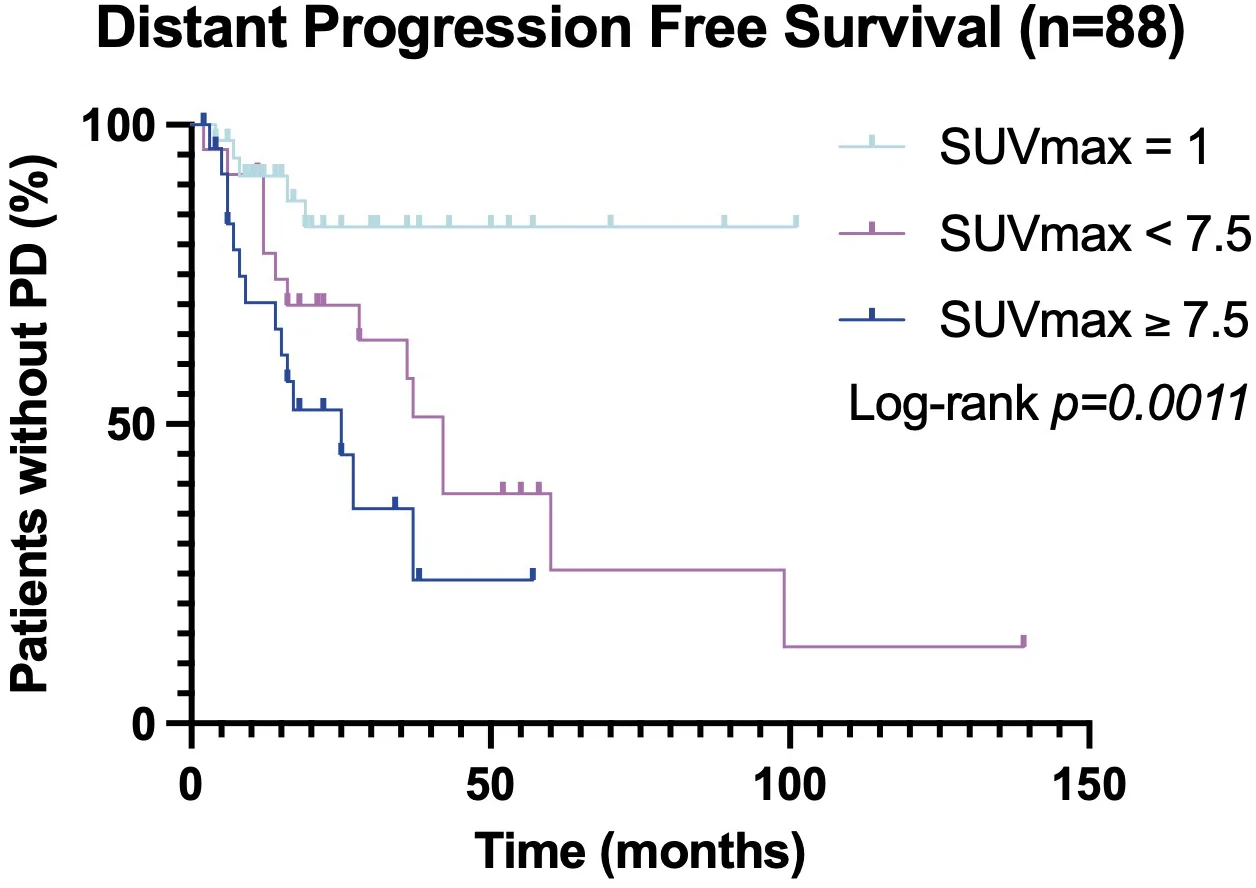
Kaplan-Meier diagram of distant progression free survival for all 88 PET/CT. PFS, progression free survival.

**Table 3:** Comparison of SUVmax thresholds and diagnostic metrics in post-treatment PET/CT studies for HNSCC.

Study	SUVmax threshold	Sensitivity (%)	Specificity (%)	PPV (%)	NPV (%)	Clinical notes
Current Study	≥1	84.9	60.0	56.0	86.8	High SUVmax associated with worse PFS; intermediate uptake also prognostic
Kim et al., 2016 (10)	≥4.4	90.0	83.8	45.0	98.3	Predictive for early recurrence; salvage surgery did not improve OS
Ito et al., 2014 (11)	≥6.1	67.0	88.0	NR	NR	SUVmax >6.1 predicted poor OS (12.1 vs. 44.6 months)
Dondi et al., 2024 (14)	Not specified	NR	NR	NR	NR	Used MTV/TLG instead of SUVmax; significant for OS and PFS
Zhong et al., 2020 (12)	Qualitative (Hopkins, Deauville)	~85.0 (avg)	~86.8 (avg)	~85.0	~85.6	Timing beyond 12 weeks improved interpretability and accuracy
Yao et al., 2007 (8)	≥3.0	100	84.2	62.5	100	Only nodal status; false positives in some cases caused by histologically confirmed inflammation
Benjamin et al., 2021 (22)	Deauville score (≥4)	NR	NR	100	92.3	Only nodal status; good concordance with SUVmax

SUVmax, maximum standardized uptake value; PPV, positive predictive value; NPV, negative predictive value; PFS, progression-free survival; MTV/TLG, metabolic tumor volume/total lesion glycolysis; NR, not reported.

### Effect of timing on diagnostic accuracy

When stratified by imaging interval, PET/CT scans performed >3 months post-treatment tended to achieve better diagnostic metrics, the maximum sensitivity, specificity, PPV, and NPV were all observed in >3 months groups. Specifically, using a SUVmax >1.0 threshold at >3 months yielded the highest sensitivity (90.9%; 95% CI: 62.3-99.5%), PPV (62.5%; 95% CI: 34.6-81.5%), and NPV (88.9%; 95% CI: 56.5-99.4%). Maximum specificity (78.6%; 95% CI: 52.4-92.4%) and similar PPV (62.5%; 95% CI: 30.6-86.3%) was observed with SUVmax ≥7.5 at >3 months. A similar specificity (78.1%; 95% CI: 63.3-88.0%) as with the SUVmax ≥7.5 at >3 months group was seen in early scans (0-3months) using the same threshold.

### SUVmax univariate analysis

Using only SUVmax values that were specifically stated in PET/CT reports (n=50; 56.8% of the entire dataset) univariate analysis did show an insignificantly increased hazard ratio (HR: 1.1; 95% CI: 0.97-1, 23). Compared to that using all available data points with an assumed SUVmax of 1 as described previously yielded a significantly increased hazard ratio (HR: 1.19; 95% CI: 1.1-1.28). This was also the case for a more moderately estimated SUVmax of 2.2 for all non-pathological PET/CTs, the lowest specifically recorded SUVmax value in the pathological groups (HR: 1.2; 95% CI: 1.1-1.3).

### Multivariate prognostic analysis

Multivariate Cox regression analysis identified SUVmax (as a continuous variable) as an independent predictor of shorter PFS (HR: 1.21; 95% CI: 1.11-1.32; p < 0.001). Additional significant predictors of reduced PFS included male sex (HR: 3.45; 95% CI: 1.2-12.34) and primary tumor location in the oral cavity (HR: 6.08; 95% CI: 1.24-25.25). Other evaluated covariates—age, HPV status, tumor differentiation, and PET/CT timing—were not significantly associated with PFS in the multivariate model (**Table 4**).

**Table 4:** Results of multivariate Cox regression analysis.

Variable	Reference level	Hazard ratio	95% confidence interval
SUVmax	Continuous	1.21	1.11 - 1.32
Timing of PET/CT	Continuous	0.93	0.84 - 1.00
Age	Continuous	0.97	0.93 - 1.01
Sex Male	Sex Female	3, 45	1, 20 - 12, 34
HPV positive	HPV negative	0.62	0.18 - 1.88
HPV not tested	HPV negative	0.96	0.34 - 2.78
Location Oral cavity	Location Larynx	6.08	1.24 - 25.25
Location Oropharynx	Location Larynx	0.67	0.20 - 2.27
Location Hypopharynx	Location Larynx	1.19	0.31 - 4.38
Location Nasopharynx	Location Larynx	2.33	0.05 - 54.46
Location Nasal cavity	Location Larynx	2.23	0.46 - 8.35
Moderately Differentiated	Well Differentiated	0.96	0.23 - 6.55
Poorly Differentiated	Well Differentiated	0.59	0.12 - 4.33
Undifferentiated	Well Differentiated	0.25	0.004 - 7.58
Differentiation unclear	Well Differentiated	0.89	0.12 - 7.95

## Discussion

This retrospective study reinforces the clinical and prognostic value of post-treatment 18F-FDG PET/CT imaging in patients with head and neck squamous cell carcinoma (HNSCC). Our findings demonstrate that elevated post-treatment SUVmax is independently associated with worse progression-free survival (PFS), with the SUVmax threshold of ≥7.5 serving as a strong indicator of disease progression. Furthermore, the timing of post-treatment PET/CT—particularly when performed more than three months after treatment—affects diagnostic performance and clinical interpretation, emphasizing the need for tailored surveillance strategies.

The prognostic utility of FDG-PET/CT in post-treatment surveillance of HNSCC has been increasingly recognized. Elevated SUVmax values following definitive chemoradiotherapy (CRT) suggest residual viable tumor and correlate with inferior oncological outcomes. In our cohort, patients with SUVmax ≥7.5 had a significantly higher risk of disease recurrence or progression compared to those with lower values, consistent with previous studies.

Kim et al. (2016) evaluated 108 patients with advanced HNSCC treated with CRT and found that a post-treatment SUVmax threshold of ≥4.4 was predictive of recurrence and poor survival outcomes, with significantly lower 3-year PFS (42.9% vs. 81.1%) and OS (56.9% vs. 87.7%) in the high SUVmax group (10). Similarly, Ito et al. (2014) showed that patients with SUVmax >6.1 after CRT had significantly shorter OS (mean 12.1 months) compared to those with lower values (44.6 months) (11). While our study used a slightly higher SUVmax threshold (7.5), likely reflecting cohort differences and institutional variations in imaging protocols, the prognostic trend is consistent.

These findings underline SUVmax as a reproducible, semiquantitative measure of metabolic tumor activity that correlates with treatment response. Importantly, SUVmax is readily available in clinical practice, does not require complex post-processing, and offers immediate guidance for further diagnostic or therapeutic steps.

A crucial determinant of PET/CT interpretability in the post-treatment setting is timing. Our data shows that scans performed more than three months after CRT yield higher specificity (78.6% vs. 78.1%) and PPV (62.5% vs. 50.0%) for identifying true disease progression when comparing a threshold of ≥7.5 for 0–3 months and >3 months. This is likely, at least in part, due to resolution of inflammation and radiation-induced tissue changes, which may otherwise mimic tumor activity in early imaging. This effect was shown by Yao et al. (2007) where some, according to PET/CT, suspicious nodal lesions were histologically examined and, in some cases, found to be reactive inflammation (8).

Zhong et al. (2020) compared four qualitative interpretative PET/CT criteria (Hopkins, Deauville, Porceddu, and NI-RADS) in 106 patients and found that the accuracy of post-treatment scans improved when imaging was delayed beyond 12 weeks (12). The risk of false positives due to inflammatory uptake in early imaging is well documented and can lead to unnecessary biopsies or overtreatment.

Accordingly, NCCN (National Comprehensive Cancer Network) and EANM (European Association of Nuclear Medicine) guidelines recommend performing post-treatment PET/CT no earlier than 12 weeks after completion of radiotherapy to minimize interpretive pitfalls (4, 6). Our findings support this practice and suggest that delayed imaging, in combination with SUVmax quantification, enhances diagnostic precision and prognostic discrimination.

A novel aspect of our study is the stratification of patients into three SUVmax-based risk categories: physiological (≤1.0), intermediate (>1.0 but <7.5), and high (≥7.5). Interestingly, even patients with intermediate SUVmax values had a significantly higher risk of progression compared to those with physiological uptake. This suggests that dichotomizing PET/CT results into “positive” and “negative” with a threshold that is >1 may overlook patients with moderate metabolic activity who still harbor significant disease risk. In contrast, patients with high SUVmax values may benefit more from intensified diagnostic and therapeutic regimes compared to ones with moderate values.

This insight parallels the clinical challenge of managing equivocal PET/CT results—scans that are not definitively normal or abnormal. In such cases, integrating SUVmax with clinical findings, anatomical imaging, and HPV status (where available) may improve risk stratification and guide management decisions. For example, Marcus et al. (2014) proposed the “Hopkins Criteria, “ a five-point qualitative scoring system that improved inter-reader reliability and prognostic accuracy in the interpretation of PET/CT scans in HNSCC (13).

While SUVmax is a convenient marker, it captures only the single most metabolically active voxel and may not reflect the full disease burden, especially in large lesions with only moderately increased metabolic activity. Volumetric PET parameters such as metabolic tumor volume (MTV) and total lesion glycolysis (TLG) have been proposed as superior prognostic indicators indicators as they reflect the underlying biology more comprehensively.

In a recent study by Dondi et al. (2024), pre- and post-treatment PET/CT parameters including MTV and TLG were significant predictors of OS and PFS in patients with oropharyngeal cancer, particularly in HPV-negative tumors (14). However, a practical drawback of these measures is that they require standardized image segmentation and are less commonly used in routine clinical workflows.

Moreover, in clinical practice, semiquantitative metrics such as SUVmax are often more feasible and cost-effective. The consistent prognostic value of SUVmax across studies, including ours, supports its continued use, while encouraging future prospective research into hybrid metrics that combine SUVmax with volumetric and morphological data.

Beyond prognosis, FDG-PET/CT may play a pivotal role in guiding salvage therapy. In our cohort, patients with elevated SUVmax underwent histologic confirmation in over 70% of cases, often leading to salvage surgery or systemic therapy. This diagnostic confirmation rate underscores the potential of PET/CT to act as a triage tool, identifying patients who warrant further invasive evaluation.

However, the role of PET/CT in guiding salvage surgery remains controversial. Kim et al. (2016) noted that surgery did not significantly improve survival in patients with high post-treatment SUVmax, suggesting that aggressive disease biology may render local salvage ineffective (10). Furthermore, depending on the study, high complication rates ranging from 33-92% were observed for salvage surgery after radiation or chemoradiotherapy (15–17). As such, the use of PET/CT in this context should be balanced against patient performance status, comorbidities, and treatment goals.

Recent large-scale studies further validate our findings on the prognostic utility of FDG-PET/CT and the importance of timing and metabolic parameters in the post-treatment evaluation of HNSCC. In a cohort of over 850 patients, Clément et al. reported that FDG-PET/CT showed excellent sensitivity (98%) and specificity (89%) for detecting subclinical recurrence (SCR), with the highest detection rates observed in the first two years after treatment (18). This supports our observation that delayed PET/CT (after 3 months) enhances diagnostic accuracy, particularly in reducing false positives due to post-radiation inflammation. Similarly, Navran et al. demonstrated in a large institutional cohort that FDG-PET/CT at three months post-(chemo) radiotherapy significantly improved selection for salvage neck dissection (SND), sparing over 20% of patients from unnecessary surgery (19). Their study showed an excellent negative predictive value (NPV) of 97.5% for complete metabolic responders, underscoring the safety of avoiding invasive procedures in patients with negative post-treatment PET scans. Wakisaka et al. further emphasized that post-treatment SUVmax was an independent predictor of both overall and progression-free survival in advanced HNSCC, aligning with our results. Interestingly, they also explored the correlation of metabolic parameters with inflammatory biomarkers such as NLR and CRP/albumin ratio, pointing to a potential multi-modal prognostic framework (20). Lastly, Leclère et al. highlighted the prognostic and therapeutic impact of incorporating FDG-PET/CT into the initial staging of HNSCC (21). Their findings indicated that PET-based upstaging was associated with significantly poorer 3-year OS compared to conventional staging, illustrating that PET/CT refines both prognostic stratification and therapeutic planning even at baseline. Together, these data affirm the integral role of FDG-PET/CT not only in response assessment but also as a prognostic and decision-making tool across the HNSCC care continuum.

Our findings carry important implications for refining post-treatment surveillance strategies in HNSCC. Patients demonstrating physiological FDG uptake—defined by absence of suspicious focal activity and interpreted as post-therapeutic changes without a measurable SUVmax—had excellent PFS and no significant benefit from additional interventions. This subgroup may be considered for de-escalated follow-up protocols, potentially reducing the frequency or intensity of imaging and clinic visits.

Conversely, patients with intermediate SUVmax values (>1 but <7.5) exhibited a significantly higher risk of progression compared to the physiological group, suggesting that this subgroup should not be overlooked as “low risk” based on qualitative interpretation alone. These results advocate for a tiered surveillance model: high-intensity follow-up and early biopsy for patients with elevated SUVmax, medium intensity follow up, for example via panendoscopy or shortened imaging intervals, for intermediate SUVmax values and reduced diagnostic burden for those with physiological uptake. This approach could optimize resource utilization and reduce patient burden without compromising oncologic safety.

Several limitations merit discussion. First, the retrospective, single-center design may limit generalizability. While SUVmax values were extracted uniformly, variations in scanner type, reconstruction algorithm, and imaging protocol may introduce bias. Second, not all patients had HPV status documented, limiting stratified analysis for this important prognostic factor. Third, the SUVmax threshold of 7.5 was determined based on the median of our cohort and may not be universally applicable. External validation in larger, prospective cohorts is needed. Although it should be noted that, as discussed by Wahl et al. (2009), absolute SUVmax threshold values are sub-optimal measures due to technological advances over time. Newer PET/CT scanners tend to have considerably higher spacial resolution which results in smaller voxels that are more prone to noise. That makes it more likely to have high outlier values. Higher spacial resolution also results in higher voxel count for a certain volume also giving more opportunities to find a high outlier. Since the SUVmax, by design, represents the single highest SUV value in a volume it is highly sensible to increases in noise (5). As a result, SUVmax thresholds should be viewed in consideration of the time at which they were published. Fourth, our study did not incorporate volumetric parameters such as MTV or TLG, nor did we evaluate standardized qualitative scores like Deauville or NI-RADS, which may offer additional prognostic insight. Fifth, the number of recorded deaths was rather small resulting in very limited capabilities for overall survival analysis. Sixth, the results of the multivariate Cox regression analysis have to be evaluated critically because the number of covariates introduces increased statistical error rates especially given the comparatively low number of progression events (33). Seventh, most patients in our cohort (86.4%) had advanced SCC (stage III or IV) limiting the generalizability for patients with stage I and II SCC. Lastly, while histologic confirmation was available in most PET-positive cases, the lack of standardized biopsy protocols may introduce verification bias. Future studies should adopt a uniform approach to correlating PET/CT findings with pathology and long-term outcomes.

The clinical implications of our findings are substantial. Post-treatment PET/CT with SUVmax evaluation should be considered a standard component of advanced HNSCC surveillance. A tiered approach based on SUVmax (physiological, intermediate, high) may better inform clinical decision-making than dichotomous interpretation. Patients with elevated SUVmax may benefit from intensified follow-up, early biopsy, or salvage therapy, while those with physiological uptake may safely avoid invasive interventions.

## Conclusion

Our findings reinforce the prognostic utility of post-treatment FDG-PET/CT in patients with advanced HNSCC, demonstrating that higher SUVmax values are independently associated with worse progression-free survival. The timing of imaging also influences diagnostic accuracy, with improved performance metrics observed beyond three months post-treatment. These results support the integration of FDG-PET/CT into routine surveillance strategies and may guide personalized post-therapeutic decision-making. Prospective validation and incorporation of advanced PET metrics remain critical next steps to enhance the role of functional imaging in head and neck oncology.

## Data Availability

The datasets used and/or analyzed during this study are available from the corresponding author on reasonable request.
